# Radiation therapy plan checks in a paperless clinic

**DOI:** 10.1120/jacmp.v10i1.2905

**Published:** 2009-01-27

**Authors:** R. Alfredo Siochi, Edward C. Pennington, Timothy J. Waldron, John E. Bayouth

**Affiliations:** ^1^ Department of Radiation Oncology University of Iowa Hospitals and Clinics Iowa City IA 52242 U.S.A.

**Keywords:** electronic medical record, quality assurance, treatment planning system, record and verify system

## Abstract

Traditional quality assurance checks of a patient's radiation therapy plan involve printing out treatment parameters from the treatment planning system and the “record and verify” (R&V) system and visually checking the information for one‐to‐one correspondence. In a paperless environment, one can automate this process through independent software that can read the treatment planning data directly and compare it against the parameters in the R&V system's database. In addition to verifying the data integrity, it is necessary to check the logical consistency of the data and the accuracy of various calculations. The results are then imported into the patient's electronic medical record. Appropriate workflows must be developed to ensure that no steps of the QA process are missed. This paper describes our electronic QA system (EQS), consisting of in‐house software and workflows. The EQS covers 3D conformal and intensity modulated radiation therapy, electrons, stereotactic radiosurgery, total body irradiation, and clinical set ups with and without virtual simulation. The planning systems handled by our EQS are ADAC Pinnacle and Varian FASTPLAN, while the R&V systems are LANTIS and VARIS. The improvement in our plan check process over the paperless system is described in terms of the types of detected errors. The potential problems with the implementation and use of the EQS, as well as workarounds for data that are not easily accessible through electronic means, are described.

PACS numbers: 87.55.Qr, 87.55.tg, 87.55.tm

## I. INTRODUCTION

An increasing number of radiation oncology clinics are transforming into paperless departments with the adoption of commercially available technology for electronic data interchange (EDI) and electronic medical records (EMR). Regulatory agencies are also driving the trend towards electronic health records (EHR) and, eventually, physician reimbursements may depend on the use of an EHR system.^(1)^

In radiation therapy, record and verify (R&V) systems have evolved into databases that include not only treatment machine parameters but also scheduling, images, assessments, document import, and Health Level 7 (HL7) support. New software versions continue to add support for the flow of digital information through our clinical processes. As a result, clinics have reported improvements in patient safety,^(2)^ data security and accessibility.^(3)^

However, these systems do not support the needs that are specific to particular clinics that have to develop custom software to improve workflow efficiency.^(4)^ ^,^ ^(5)^ Current R&V or EHR systems still do not address the issues of logical consistency of data that enter the system from various sources, nor do they examine the accuracy of treatment data relative to the physician's intent. Data integrity, especially where changes in treatment data may be updated in only one source, is still a concern even for systems that directly link treatment plan parameters to the R&V database. The potential for changing data in one application without examining its effects in a different application (e.g. modified dose distributions) still exists.^(6)^

To address these shortcomings, we have developed an electronic radiation therapy plan Quality Assurance (QA) system (EQS) that consists of software modules incorporated with well documented processes and policies. Prior to the implementation of the EQS, we would either print out, or electronically display, the relevant plan parameters from the treatment planning system (TPS) along with the corresponding data from the R&V system, and visually confirm that the treatment parameters were identical. A number of shortcuts were used to validate data integrity, especially for Intensity Modulated Radiation Therapy (IMRT) treatments with many segments. With the EQS, all treatment parameters (including each leaf position of every single segment in IMRT plans) are exported from the R&V system, and are compared to the data used by the planning system for its display of plan information and dose distributions. The EQS performs a hierarchical match to find corresponding data elements and alerts the user if it finds any errors. The EQS also examines the treatment data for consistency with the physician's prescription. It checks for any interlock or illegal parameter combinations to ensure that the LINAC is able to deliver the treatment. Once these tests are passed, the EQS is used as a second independent MU calculation check. When the fields are approved (and locked) for treatment, a snapshot of the checked parameter set is saved as the reference data for a final check of the database; this is to ensure that no inadvertent modifications were introduced.

This paper will describe the software modules and the processes that make up our EQS, along with limitations and recommendations resulting from our use of the system.

## II. MATERIALS AND METHODS

### A. EQS Modules

The modules in our EQS address three major steps: plan quality assessment, TPS parameter export, and data integrity verification between the R&V and TPS.

The quality of the treatment plan is evaluated using the Computational Environment for Radiotherapy Research (CERR),^(7)^ an independent plan review program developed in MATLAB (Mallinckrodt Institute of Radiology, Washington University Medical Center, St. Louis, MO). CERR displays Dose Volume Histograms (DVH) and can independently compute them from the exported Radiation Therapy Oncology Group (RTOG) plan data. It also displays dose distributions in a continuous scale color‐wash overlaid on patient images. This display enables physicists to find objectionable dose distributions that may not have appeared in the isodose lines in Pinna‐cle3 (Philips Healthcare, Andover, MA).

**Figure 1:**
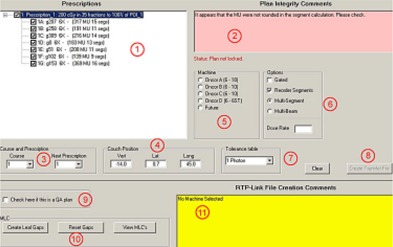
Screen shot of LEX. The numbered areas correspond to the following descriptions: (1) Prescription and beam data are read directly from Pinnacle's data file; (2) The transfer software looks for conditions that do not follow our clinical practice; (3) Pinnacle has no provision for course or prescription number; the operator can select a course number and prescription number in order to create a unique ID for each beam; (4) Pinnacle has no fields for table position; the operator can edit the default values determined by LEX from the isocenter; (5) The Siemens ONCOR linear accelerators in our clinic have matched beam energies, so the operator must select a machine from the list; (6) The operator can choose to have the beams gated, re‐order the segments in multi‐segment beams to minimize the delivery time, and/or convert multi‐segment beams into a series of individual beams to handle gated IMRT; (7) Pinnacle does not provide tolerance tables, so the operator must choose the proper one from the drop down list; (8) The button to create the RTPLINK file is disabled until all errors are cleared; this prevents the operator from sending incomplete data; (9) By checking the QA box, beams will be created that have a unique “QA” field ID; (10) These buttons control MLC options such as adding a 2 mm gap between closed leaf pairs that are blocked by the collimators; (11) Unresolved errors are displayed in this box. Once all the errors have been cleared, the button to create the transfer file is enabled.

The transfer of plan parameters to the R&V database LANTIS (Siemens Medical Solutions, Concord, CA) is accomplished with an in‐house application called LEX (Fig. 1), which reads data from the Pinnacle3 planning system and creates an RTP‐Connect file^(8)^ that can be imported into LANTIS. LEX performs a number of checks on the planning data to ensure that they are compatible with the requirements of the delivery system and the R&V database, flagging the user to fix any inconsistencies.

**Figure 2:**
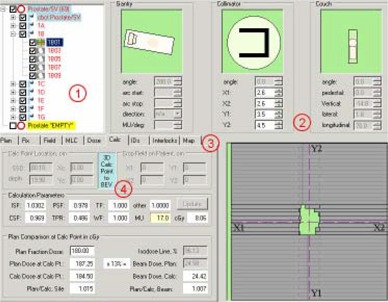
Screen shot of the RTP‐Filter. The numbered areas correspond to the following descriptions: (1) The prescriptions, beams and segments can be selected by the operator; (2) The position parameters (gantry, collimator, couch, MLC leaves) of each segment can be viewed in the panels in this section; (3) More information about the plan, the selected prescription, field and MLC, the dose, beam IDs, interlocks and intensity maps can be reviewed by selecting the appropriate tabs; (4) Pinnacle plan to LANTIS field comparisons are done when the user performs a dose calculation using the LANTIS data; calculation parameters for flash and effective depths can also be modified in this tab.

**Figure 3:**
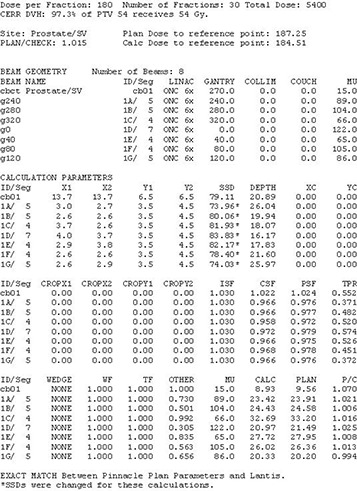
Sample MU check report from LANTIS. (Note: patient identifiers and physicist's signatures were removed). The calculation uses the field parameters from LANTIS, and the patient SSD and depth information from Pinnacle as a starting point. The depths are modified by the physicist to account for the 3 dimensional nature of the heterogeneities and patient contours. All dosimetric parameters were taken from an electronic version of the dosimetry calculation book. The parameters in the report are effective parameters, accounting for the variations among the segments for each beam.

**Figure 4:**
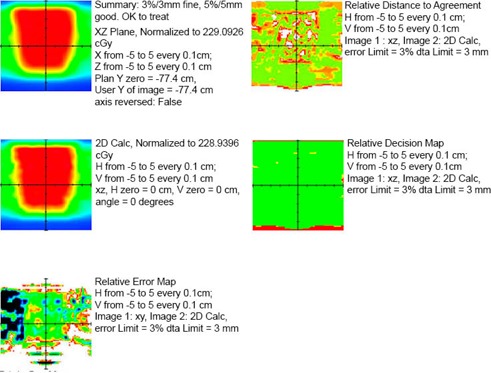
Sample IMRT QA report from LANTIS. (Note: patient identifiers and physicist's signatures were removed.) The QA method in this report is the 2D calculation using a ray trace through a solid water phantom. All SSDs and depths are calculated from intersections of each ray line with the phantom surface planes. This is done for each point on the 2mm×2mm grid on the coronal plane through isocenter. The resulting calculated “virtual film” (middle left) is compared against the corresponding 4mm×4mm Pinnacle calculation (top left) in order to determine the error (bottom left), distance to agreement (top right), and decision (middle right) maps. The calculated data were interpolated onto a common 1mm×1mm grid.

Once all plan parameters are in LANTIS, the LANTIS data can be exported as an RTP‐Connect file. Another in‐house application, the RTP‐Filter (Fig. 2), reads this R&V data file and compares it against the Pinnacle plan parameters. The RTP‐Filter informs the user of any differences as well as any logical inconsistencies in the data. It also performs an independent MU check and creates QA reports (Fig. 3 and Fig. 4).

Since additional interaction can occur with the database, the RTP‐Filter is also used to compare various states of the database. This is achieved by comparing two RTP‐Connect files: an initial export (IE) file, exported right after a successful comparison with the plan data, and a final export (FE) file, exported just prior to treatment.

While it might seem possible to create a single application that performs all steps, the modular structure provides extra safety. RTP‐Filter and LEX were written by different programmers (co‐authors of this report, Alf Siochi and Ed Pennington, respectively) and the code bases are completely independent (visual basic.NET and visual basic 6.0, respectively). It is less likely that the same bug will be present in both applications. Additionally, clinical workflows are iterative, with different modules being used at different stages. Finally, not all clinical processes can use the same workflow, and separating functions into modules allows one to modify or create new modules for those parts of a process that vary.

## B. LEX

### B.1. Overview

LEX transfers data from the planning system (Fig. 1.1) to the R&V system. While commercial applications exist that will perform this task, we have found that these applications omit data, leading to significant manual entry and editing. Our custom‐built transfer program, LEX, supplements the planning data with the additional information needed to create a complete data package with minimal manual entry. Additionally, LEX can compare the plan to specific clinic protocols, warning the operator of inconsistent data prior to the actual transfer (Fig. 1.2 and Fig. 1.11). Such integrity testing significantly reduces the chance of transferring unintended (or inaccurate) data to the R&V system.

### B.2. Pinnacle Data Access

LEX runs on the dosimetrists' Windows platform PC in a read‐only mode, and does not make any modifications to data on the planning system. The Pinnacle planning system stores all the pertinent plan information in simple ASCII text files. LEX uses the File Transfer Protocol (FTP) in the background to copy these text files from the Pinnacle server to the dosimetrists' PC. LEX reads the local copies of the text files and extracts the information that is required for the R&V system (Fig. 1.1). LEX will translate the Pinnacle nomenclature to valid LANTIS field names where necessary. Once processed, the data are written to a separate file in RTP‐Connect format for import into the LANTIS R&V system.

FTP Access to the class A Pinnacle network is granted only to a specific list of Internet protocol (IP) addresses within the class B hospital network. The configuration was reviewed by our IT staff and deemed HIPAA compliant.

### B.3. Additional Treatment Information

LEX reads the Pinnacle patient index and allows the dosimetrist to select the proper patient, plan, trial, prescription and beams to transfer. LEX prompts for additional data before the plan transfer file is created (course number, prescription IDs [Fig 1.3], couch positions [Fig 1.4], a machine ID [Fig 1.5], dose rate [Fig 1.6], and a tolerance table [Fig. 1.7]). These data are not part of the Pinnacle plan but are required for the R&V system. The operator selects the additional data either from drop down menus or text boxes. LEX will not create the transfer file until all required parameters have been entered correctly. For example, the operator will be warned if the selected treatment machine does not offer a beam energy for a field in the plan (Fig 1.11). Many such tests are performed to prevent incidental errors.

The operator must choose whether beams with multiple control points (IMRT or forward planning fields) are transferred as multi‐segmented fields or individual beams (i.e., one beam per Pinnacle control point) (Fig 1.6). Another option button determines whether or not the beam is gated. The operator can also re‐sequence the MLC segments in order to minimize delivery time. The operator can request that a 2 mm gap be placed between MLC leaves that are covered by the overlying jaws (Fig 1.10) to reduce MLC collisions.

### B.4. Plan Integrity Checks

LEX tests for plan consistency against clinical practice and will, for example, warn if the isodose distribution is not normalized to the prescription point. LEX consistency tests further ensure that all beams were calculated with the same model (heterogeneous or homogeneous) and verify that beams under a specific prescription have the same calculation point. At our institution, IMRT plans are generally prescribed to “Set Monitor Units”. Therefore, LEX issues warnings for IMRT plans set to other prescription options. While these tests do not necessarily indicate errors in the plan, they point out abnormal behavior that might indicate unintentional actions. The operator has the choice as to whether or not to continue the transfer with the suspect data for these types of warnings.

### B.5. Beam Integrity Checks

LEX checks each beam in the plan to ensure clinical practice conformity. For example, we have chosen to use only 60 degree virtual wedges. Although Pinnacle allows other virtual wedge angles and the accelerator will accept them, wedges other than virtual 60 degree will not pass LEX. Beams with unusually high or low monitor units are flagged, as well as beams that use too few monitor units for virtual wedges. The SSDs and depths of each field are tested to ensure they fall within normal boundaries. LEX ensures that the monitor units for individual control points are integer values, since our R&V system will not accept fractional MUs. Our Siemens ONCOR linear accelerators utilize MLCs that replace the X jaws. Pinnacle has a separate X jaw parameter that should be set to the maximum MLC opening, and LEX verifies this. Other administrative controls can be added as needed.

### B.6. Limitations

It is important for individuals using these tools to recognize their limitations. LEX is not an editor. LEX is designed primarily as a plan transfer tool. If LEX uncovers inconsistencies in the plan data, it prompts the user to correct the problem on the planning system (Fig. 1.2). Once the inconsistencies have been corrected on the planning side, LEX can be used again to test and transfer the plan data. LEX does not independently calculate the monitor units per beam. LEX cannot interpret a prescription to see if it makes clinical sense, or interpret the dose distribution to see if it meets the objectives of the physician. Human intervention is still critical to the QA process.

Each time we encounter a situation where data was accepted for treatment that was either incorrect or unintended, we review the workflow and electronic testing mechanisms to ascertain whether additional checks are warranted. Many of the features in LEX were added for this reason. LEX's purpose is to identify irregularities in the plan and beam data before that data is entered into the R&V system. If LEX cannot find fault with the data, it is accurately transferred.

## C. RTP‐FILTER

### C.1. Overview

The primary purpose of the RTP‐Filter is to verify that all the fields in the R&V system match those of the plan. It can compare RTP‐Connect files exported from the R&V system to the data files used by the Pinnacle planning system. It can also compare two RTP‐Connect files that were exported at different times to verify that no changes occurred to the data in the R&V database. It also tests the data in an RTP‐Connect file to ensure that they are logically consistent (e.g. beam energies match the physician's prescription). Finally, it provides an independent MU check by calculating the doses resulting from the R&V beam parameters and comparing them against the Pinnacle doses.

### C.2. Plan Parameter Comparison

When comparing Pinnacle Plans and R&V data, the RTP‐Filter uses a minimum deviation matching strategy to map the R&V treatment fields (Fig. 2.1) to the Pinnacle beams. For a given R&V treatment field, the RTP‐Filter iterates through all unmatched Pinnacle beams to find the beam with the least deviation from the R&V field. If it finds a beam with a deviation of zero, it stops the current iteration and proceeds to the next R&V field. The deviation is the sum of the absolute values of the discrepancies between positional parameters and the sum of importance values for non‐positional parameters:

(1) $$\Delta =|G_{R}-G_{P}|+|C_{R}-C_{P}|+|T_{R}-T_{P}|+I+\sum_{i}S_{i}$$

(2) $$I=|E_{R}-E_{P}|+W+100M+\frac{L|N_{R}-N_{P}|+|U_{R}-U_{P}|}{10}$$

(3) $$S_{i}=\sum_{j}|Yj_{Ri}-Yj_{Pi}|+\sum_{k}|Xk_{Ri}-Xk_{Pi}|+\frac{\left|\mu_{Ri}-\mu_{Pi}\right|}{10}$$

where Δ=deviation, the subscripts R and P stand for R&V and Planning System, respectively, G=gantry, C=collimator, T=couch, I=non‐positional importance function, $S_{i}=\text{deviation for the ith control point}$, E=Energy, W=wedge comparison function, M=modality comparison function, L=number of multi‐leaf collimator(MLC)leaves, N=number of control points, U=monitor units for the beam, Yj=position for the jth jaw(IEC jaws X1,X2,Y1,and Y2), Xk=position for the kth leaf of the MLC, and $\mu_{\mathrm{03BC}}=\text{monitor units for the ith control point}$. The comparison functions are equal to 1 when the parameters being compared do not match, and are zero when they match (e.g. W=1 when WR≠WP). The pinnacle beam with the minimum deviation is then considered as the match for the LANTIS field. Once a pinnacle beam is matched, it is removed from the matching process. Several iterations proceed until all R&V fields have been matched to all planning beams. If the total deviation from all matches is zero, then we have an exact match for the entire plan.

The quantity $\sigma S_{i}$ is evaluated only when the R&V field and the Pinnacle beam being considered have the same number of control points and total MU (i.e. the expressions involving N and U go to zero). The control point deviation also goes through a minimization process where control points of an R&V multi‐segmented field are matched against the control points of a Pinnacle beam. The index i in Equation 1 refers to the control point index of the R&V multi‐segmented field and that of the matching Pinnacle beam.

A similar comparison process is used for comparing the initial and final export files from the R&V system. An IE field is compared against an FE field to determine if they belong to the same beam. That is to say they should have the same values for gantry, collimator, arc start and stop angles, arc direction, arc MU per degree, energy, modality and beam modifiers such as bolus, wedges, and electron applicators, and a couch angle difference that is within the difference defined by the tolerance table. If the fields belong to the same beam, their MLC leaf positions, jaw positions, MU, treatment site and field IDs are then compared. The deviation will be equal to the sum of the absolute values of the differences of all leaf and jaw positions, plus a fixed value of 0.00001 each time the MU, site and field IDs are different. The selected field match will be the item with the minimum deviation. If there is no FE field that belongs to the same beam as the IE field, then an attempt is made to match on the basis of field ID. If no matching field is found, the user is notified of the error in the report.

#### C.3. Logical Consistency

##### C.3.a. Physician's Prescription

There are data elements in the physician's prescription that are manually entered into the R&V system. The only check of such data occurs by comparing them against the rest of the data that was imported from the planning system. For example, if the physician chooses 6X as the modality, then all the treatment fields that belong to that prescription must use 6 MV X‐rays. The modality item is implemented in LANTIS as a drop‐down box, and the wrong item is occasionally chosen. Fortunately, LANTIS exports this item as a string in the RTP‐Connect file, and the RTP‐Filter parses the modality string. If the RTP‐Filter finds energies and particles other than those defined in the modality, it alerts the user.

Sometimes the total dose and fraction dose are manually entered in LANTIS. If physicians decide to change the number of fractions, LANTIS only lets them edit the total dose. Hence, when the RTP‐Filter compares a Pinnacle plan with the R&V treatment site data, it determines the number of fractions in the R&V system and compares that against the number of fractions in Pinnacle. The fraction dose is implicitly checked during the monitor unit calculation check (see Section II.C.4), and the user is warned if the dose calculated using the R&V data differs from the Pinnacle calculation point dose by 3% or more.

The individual doses assigned to each R&V treatment field are populated by LEX, and checked by the RTP‐Filter. The user is alerted if the sum of the individual doses does not match the fraction dose of the prescription. These treatment field doses are important for the dose tracking that the R&V system uses to notify the therapists if they are about to exceed the prescription dose, or if they are to perform some action at a particular dose.

Finally, the RTP‐Filter calculates the isodose line from the fraction dose of the R&V system and the dose reported by Pinnacle at the prescription point. Unfortunately, the isodose line is not a standard item that is exported in the RTP‐Connect format. The physicians usually write this value in a field in the R&V database that does not get exported. The physicist must visually check the prescription notes in the R&V database against the value reported by the RTP‐Filter.

##### C.3.b. Interlocks

Certain values or conditions for treatment machine parameters are not allowed. For example, Siemens' MLC leaves must not interdigitate, and each Y jaw must not be more than 5 mm from the edge of the first open leaf pair. There are restricted ranges of motion for table angles, table translational positions, collimator angles, jaws and MLC leaves. There are also requirements on jaw positions for a given electron applicator. The RTP‐Filter inspects all the data and alerts the user if any of these interlocks will be tripped when the therapists attempt to download the treatment fields to the linear accelerator.

##### C.3.c. Translational Table Positions

All treatment fields that use the same isocenter should have the same values for the table's vertical, lateral and longitudinal positions. The RTP‐Filter assumes that all fields of a given prescription share the same isocenter and warns the user if these fields do not have the same translational table positions. If this error is not corrected, the fields can not be grouped into an auto‐sequence on the delivery machine.

##### C.3.d. Field ID Conventions

Field IDs in LANTIS must be unique and are limited to 5 alphanumeric characters. We use a convention for determining Field IDs that allows one to map them to unique block codes. This is important for auxiliary beam modifying devices like the MODULEAF (Siemens Medical Solutions, Erlangen, Germany) that rely on block codes to determine the field shape to be downloaded. For MODULEAF plans, the RTP‐Filter will check that all field IDs are consistent with the conventions in our clinic and that all block codes have been correctly mapped to field IDs.

##### C.3.e. Setup Information

The patient's chart often includes setup fields that are used solely for the purpose of port films. These setup fields should not have any assigned dose as they will contribute to the dose tracking; the RTP‐Filter alerts the user if setup fields have dose. The RTP‐Filter attempts to recognize a field that could be a setup field by looking for key words in the field name (e.g. “drr”, “setup”, “set‐up”, “set up”) or recognizing typical setup field parameters (gantry=0 or 90 degrees or 270 degrees with a field size of $10\times 10\;{\text{cm}}^{2}$). If it encounters these indicators, it prompts the user for confirmation.

##### C.3.f. SRS

The Varian FASTPLAN planning system for SRS does not have any planning data files that are exposed to the users. Plan parameters are exported as an RTP‐Connect file, but the parameters are modified to match the coordinate systems of our machines prior to import into LANTIS. The RTP‐Filter can not compare LANTIS entries to FASTPLAN parameters directly. The only check that the user can perform is a visual inspection, but the process is eased by creating a summary report. Every stereotactic arc must meet certain requirements (e.g. block code tray = “STEREO”, arcs avoid gantry and table collisions). The RTP‐Filter verifies that all these conditions are met, and notifies the user of the plan's logical consistency status. Additional information regarding the isocenter is included in the field setup note with a well‐defined string format. The RTP‐Filter parses this note to determine the isocenter coordinates for each field. It groups all arcs that share the same isocenter, and analyzes the arc configuration to determine if it is one of our standard arc sets. The name of the standard set is written next to each isocenter in the comments section of the report. Additional checks inform the user if a non‐stereotactic field is within a stereotactic prescription or vice versa.

##### C.3.g. Total Body Irradiation (TBI)

Since TBI treatments always use the same field parameters (except for SSD and MU), the RTP‐Filter checks that the LANTIS fields match our standard settings. The MU for each field is checked by using the SSD information in LANTIS, and the LANTIS SSD information is checked against the measurements taken on verification day. The MU in LANTIS is multiplied by various correction factors taken from TBI dosimetry tables to determine the dose at mid‐plane at the level of the umbilicus. The RTP‐Filter alerts the user if this value is different from the prescribed dose by more than 3%. In this case, the logical consistency check is also a plan parameter check, since the plan must match a standard template.

#### C.4. MU Checks

##### C.A.a. 3DCRT Point Calculations

The quantity calculated is the dose. This is done with an electronic version of our dosimetry data book that is loaded when the RTP‐Filter starts. Using the depths from the pinnacle plan, the Tissue Maximum Ratio (TMR) is calculated for a zero field size (TMR[0]), and the Scatter Maximum Ratio (SMR) is calculated using a modified Clarkson algorithm that considers rectangular integration elements, one for each of the MLC leaf pair apertures. The phantom scatter factor (PSF) and the collimator scatter are also determined by integration over the same rectangular elements. If there is flash, the user specifies a cropping distance that reduces the openings of the MLC leaf pair apertures for the SMR and the PSF The CSF data are scaled so that they are consistent with a detector's eye view calculation. The location of the calculation point is transformed from the patient coordinate system to the beam's eye view system (Fig. 2.4) in order to determine off‐axis ratios (OAR, within the field) or penumbra (PF) and/or leakage (L) contributions to the dose (outside the field or at a certain distance from the field edge within the field). The inverse square factor (ISF) is determined from the source to calculation point vector (in the beam's eye view system) using the vector component along the central axis as the distance (usually 100 cm for isocentric treatments) in the denominator of the ISF expression (the distance in the numerator is the calibration point distance of $100\;\text{cm}+d_{\text{max}}$). Finally, wedge factors (WF) and wedge off‐axis ratios (WO A) are determined. All the fluence related factors are multiplied by the monitor units to create a fluence factor (FF):

(4) FF=MU×OAR×WOA×WF×ISF

WF is depth dependent for physical wedges, but for virtual wedges this is ignored and set to 1. For points inside the field, the dose becomes

(5) $$D_{in}=FF\times \bar{CSF}\times PSF(0)\times [TMR(0)+\bar{SMR}]$$

The bars over CSF and SMR indicate that the values are integrated over the MLC shape in question. For points outside the field or points inside the field but in the penumbra region, a penumbra function (PF) provides the dose variation as a percentage of the value at the center of a given field size. This function is field size and depth dependent. Hence, an estimate of an equivalent field size, $S_{e}$, is determined by taking the equivalent square for a rectangle whose area is equal to that of the area exposed by the MLC aperture and whose width is equal to the average width of all non‐zero MLC leaf openings. The dose at the center of this field is given by

(6) $$D_{s}=\frac{FF}{OAF}\times CSF(S_{e})\times PSF(0)\times [TMR(0)+SMR(S_{e})]$$

The dose at the calculation point, if inside the field near the penumbra edge, is then given by

(7) $$D_{in,pen}=PF(S_{e})\times D_{s}$$

If the point is outside the field, then leakage dose is added:

(8) $$D_{out}=PF(S_{e})\times D_{s}+TMR(0)\times MU\times L$$

The factor L depends on whether the calculation point is blocked by one or two layers of jaws. In all the depth dependent quantities in equations 4 through 7, the depth is that of the calculation point projected to the beam's central axis assuming a flat phantom whose surface is perpendicular to the central axis. The dose contribution from all fields is summed to get the total dose at the calculation point. The dose contribution for each beam is also determined, so comparisons with Pinnacle can be done on a beam by beam basis as well (Fig.3).

##### C.A.b. IMRT

For single point calculations, the IMRT calculation method is identical to that for 3DCRT, since we use step and shoot IMRT. The dose is calculated for every single segment (odd index control points) according to Section II.C.4.a. Depths are taken from the patient information in the Pinnacle plan.

For 2D dose distributions, a user‐specifiable rectangular slab phantom geometry and calculation plane are used. We typically use an isocentric coronal plane with a resolution of 2mm×2mm. An independent ray tracing algorithm within the RTP‐Filter determines the depth and beam's eye view coordinates for the entire calculation plane. The single point calculation is repeated at each point to yield the 2D dose distribution. This is exported as a binary file that can be read by our in‐house film densitometry application. The IMRT QA process proceeds as if the data came from a film measurement of the dose distribution to a solid water phantom of the same dimensions. This “virtual film” is compared to the Pinnacle dose distribution resulting from the patient's plan being hybridized with a solid water phantom of the same dimensions. If the results using the virtual film are out of tolerance, the IMRT QA is performed with an ion chamber and Kodak EDR2 film. The IMRT QA report that results from the comparison is imported into the LANTIS database (Fig. 4).

##### C.A.c. Electrons

Electron cutouts that are drawn in Pinnacle using the “manual” method will have their contour coordinates listed in the “plan.Trial” file. (Automatic blocks can be converted into a “manual” contour.) The RTP‐Filter can import these contours and estimate the dimensions of inscribed and bounding rectangles. For clinical cutouts, the RTP‐Filter will prompt the user for these dimensions. These dimensions are averaged, and the cutout factor is the square root of the product of the cutout factors of L×L and W×W squares, where L and W are the length and the width of the average rectangle, respectively. The cutout factors are read from an electronic version of the dosimetry book. The RTP‐Filter also prompts the user for the isodose line of the prescription, since the RTP‐Connect file normally does not contain this information. All our prescriptions assume that the prescription point is at the depth of $d_{\text{max}}$, and that the isodose lines are given as a percentage of the dose at this point. Information about the SSD and bolus are read from the RTP‐Connect file to perform electron inverse square corrections. The MUs are then calculated and compared against the MUs calculated by the dosimetrist who enters them into the R&V system. If the MUs differ by more than 2%, the error is flagged. An EXCEL spreadsheet with the MU calculation and basic treatment parameters is created in the process. Note that a visual comparison between the actual cutout and the plan printout is done by the dosimetrist and the therapist.

#### C.5. Log File

The RTP‐Filter writes a log file that includes all the parameter comparison reports and logical consistency check results. It also includes the names of the users who ran the plan QA and the actions that they took to perform the QA. When there is any doubt about the status of a patient's plan QA, physicists consult the log files. This is useful when the physicist that did the original plan QA is unavailable, or when a physicist is trying to determine when erroneous entries in the R&V database may have occurred.

### D. Plan Check Workflow

#### D.1. Completely Paperless

**Figure 5:**
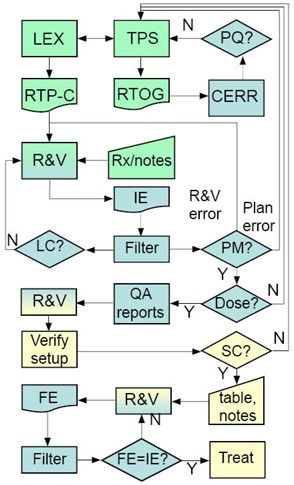
An overview of the electronic plan check workflow. The workflow starts from the TPS and ends with the treatment. Rectangles=processes or software, diamonds=decisions, trapezoids=manual input, rectangles with curved bottom=files. The boxes are color coded by function: green=dosimetrists, blue=physicists, yellow=therapists. The abbreviations are as follows: TPS=treatment planning system(Pinnacle), LEX=in‐house plan transfer software, RTP‐C=RTP‐Connect file, R&V=Record and Verify(LANTIS), Rx=prescription, IE=initial export, Filter=RTP‐Filter(an in‐house application for plan checking), LC=logically consistent, PM=Plan matches R&V, PQ=Plan quality is acceptable, SC=patient setup consistent with the plan, FE=final export.

Fig. 5 shows the workflow for most of our treatments. Once physicians approve a treatment plan, they enter the prescription into LANTIS. The dosimetrist then reads the data from the TPS and creates a RTP‐Connect (RTP‐C) file using LEX. The file is then imported into LANTIS. The dosimetrist also exports the plan from Pinnacle in RTOG format. A LANTIS Quality Check List (QCL) item is sent to Physics to inform them that a plan is ready for review. These QCLs prompt individuals to begin their portion of the process.

The physicist imports the RTOG files into CERR in order to assess plan quality (PQ). If there are problems with the plan, it is revised until the physicist, dosimetrist, and physician consider the plan acceptable. Minor plan changes are entered directly in LANTIS, while multiple changes made in Pinnacle require LEX to create a new RTP‐Connect file.

The physicist then exports the plan from LANTIS as an RTP‐Connect file. The file is renamed with patient information and the code “IE”, and is placed in the “Initial Exports” folder. The physicist then opens the IE file using the RTP‐Filter. While the application reads the file, it examines the data for logical consistency (LC). If it detects problems, it requires the physicist to acknowledge them. It will then create a report summarizing the items that need attention. The physicist then resolves these errors, deletes the previous IE file, and exports the corrected RTP‐Connect file as the IE file.

Once the IE file is opened without problems in the RTP‐Filter, the physicist imports the Pinnacle “plan.Trial” file for the treatment site that is being checked. As the filter reads the Pinnacle data, it compares all the treatment parameters listed in Table 1 against the corresponding data in LANTIS to see if it obtains a plan match (PM). It also reads all the relevant LANTIS data for performing an independent MU check. A report summarizing the comparison status identifies any errors. The physicist, the dosimetrist, and possibly the physician, work to resolve the errors. The previous plan checking steps are repeated until all discrepancies are eliminated. The MU calculation and plan comparison report file (Fig. 3), as well as an IMRT QA report (Fig. 4), if applicable, are imported into LANTIS in the E‐Scribe (E‐Scribe Inc, Bellmore, NY) section of the patient's chart.

**Table 1:** A list of parameters and the method of checking.

*Reference Check*	*Pinnacle LANTIS*	*LANTIS LANTIS*	*Pinnacle VARIS*	*FASTPLAN LANTIS*
Modality	A	A	A	T(xrays)
Energy	A	A	A	T(6 ST)
Gantry	A	A	A	N/A
Collimator	A	A	A	T(90)
Couch Angle	A	A	A	M
Couch Lat.	N/A, L	A, L	N/C	N/A
Couch Long.	N/A, L	A, L	N/C	N/A
Couch Vert.	N/A, L	A, L	N/C	N/A
Jaws	A	A	A	T(5×5)
MLC leaves	A	A	A	T(2.5)
MU	A, L	A	A, L	M, L
Wedges	A	A	A	T(none)
Bolus	M	A	M	T(none)
e^–^ Applicator	L	A	L	T(none)
Block codes	M, L	A	M, L	T(STEREO)
Cutouts	M	N/A	M	N/A
Arc Start	A	A	A	M
Arc Stop	A	A	A	M
CB MU	A	A	N/A	N/A
CB Start	A	A, T	N/A	N/A
CB Stop	A	A, T	N/A	N/A
CB Filter	N/A	N/A	N/A	N/A
Dose Rate	(LEX: M)	A	N/C	T (0)
Gating	(LEX: M)	A	N/A	N/A
Isocenter	N/A	N/A	N/A	M, L
Interlocks	L	L	L	L
Rx Modality	L	L	L	L
Fraction Dose	L	A	L	M, L
Total Dose	L	A	L	M, L
Rx Isodose	M, L	M, L	M, L	M, L
Rx Technique	M	A	M	M
Field doses	L	A	L	L
Field ID	M, L	A	M, L	M, L
Set‐up notes	M	A	M	M
Field Names	M	A	M	M

A=Automated, M=Manual, L=Logical consistency, T=Template value, CB=cone beam, N/A=not applicable, N/C=not checked. L and T are also automated. The top row indicates the reference system and the second row lists the system that is being checked. For FASTPLAN, the value in the parentheses following T indicates the expected value. TBI checks are all by template, except for lung blocks (Manual), SSD (Manual) and MUs, which are calculated (Logical consistency). The requested CB Filter is documented in the LANTIS notes and selected at the LINAC (Manual).

With an acceptable plan successfully transferred into LANTIS, the physicist completes the “Planning Physics Check” QCL and creates a “Therapist Check” QCL. The therapists review the patient's chart to ensure that all plan check documents are present and that text annotation on the Digitally Reconstructed Radiographs (DRRs) match the data in LANTIS. (Parameters such as gantry, collimator, and couch angles, as well as jaw positions and SSDs are text blocks on the DRR exported from Pinnacle for each treatment beam.) The therapists ensure that all the required elements are in place for verification day. On verification day, the therapists position the patient and acquire verification images. If the setup is consistent with the plan (SC in Fig. 5) and the physicians approve the images, the therapists enter table translational positions for each beam in LANTIS. They also enter any modifications to field setup notes and send a “Chart Check” QCL to the physicist. However, if the therapists need to modify other parameters, they call the dosimetrist. The modifications are made in the treatment plan and the physician must review the resulting dose distributions. The “Planning Physics Check” QCL is sent to the physicist and the QA process is repeated.

Once the plan is accepted by the therapists, the physicist responds to the “Chart Check” QCL by exporting the RTP‐Connect file as a final export (FE) file into the “Final Exports” folder. The FE file is then opened in the RTP‐Filter and compared to the IE file. Since the IE file provides a snapshot of a known good point of the database and it accurately represents the treatment plan parameters, it can be used as the reference file for plan comparison. If the FE file contains no errors, the physicist approves the treatment beams in LANTIS, locking them and preventing further modification.

#### D.2. Modified Workflows

A number of our clinical processes cannot use the completely paperless workflow, since they use different V&R or planning systems that are not amenable to the process. However, we modified these processes to automate them as much as possible.

The satellite clinic uses VARIS (Varian, Milpitas, CA), so a modified version of LEX creates RTP‐Connect files that are imported into VARIS via RTP‐Exchange (Varian, Milpitas, CA). VARIS exports its data in DICOM‐RT format, so the RTP‐Filter reads the DICOM‐RT file and translates it into an RTP‐Connect file. The VARIS data is then compared to Pinnacle. QA reports are printed out for inclusion in the paper chart. The physicist who travels to the site completes the paper‐based checks, reviewing the beam parameters in the paper chart used to record the patient's treatments.

For stereotactic radiosurgery, we use FASTPLAN (Varian, Milpitas, CA). The RTP‐Connect file that it exports has to be modified due to coordinate system differences and missing information. A co‐author of this report, Ed Pennington, has written FastPlanFixer, a Visual Basic 6.0 program that reads the FASTPLAN generated RTP‐Connect file and makes these repetitive edits. The modified RTP‐Connect file is then imported into LANTIS. A separate EXCEL macro reads an electronic copy of the plan printout and uses its own dosimetry tables to independently calculate the monitor units for each arc. The checking physicist reviews the data in LANTIS and visually confirms that the prescribed dose recorded in LANTIS matches the plan dose. The physicist also confirms that all the required documentation is present. The physicist exports the plan from LANTIS as an RTP‐Connect file which is checked with the RTP‐Filter.

For TBI, virtual simulation (VSim Coherence Dosimetrist, Siemens Medical Solutions), and clinical setups, we do not use a planning system. In the case of Vsim, an RTP‐Connect file is created by the VSim workstation, but for TBI and clinical setups, the fields are entered into Lantis manually. MUs are calculated by a resident and entered into LANTIS. In all these cases, the RTP‐Filter can still be used for an MU check. It can also catch logical inconsistencies in the data. For VSim, field parameters can be inspected against the LANTIS data using side‐by‐side displays from adjacent Lantis and VSim workstations. For TBI, the RTP‐Filter can verify that the field parameters match our standard setup. For clinical setups, the physicist has to verify that the field parameters in Lantis match other information in the chart that was used to set up the patient.

## III. RESULTS & DISCUSSION

### A. Errors Caught by the RTP‐Filter

Prior to the implementation of the RTP‐Filter, several test patients were exported from the R&V system. Visual comparisons were made to ensure that the RTP‐Filter interpreted and displayed information correctly. The data in the exported files were changed, one parameter at a time, to see if the RTP‐Filter would catch the errors. It caught every one.

In clinical use, we have caught discrepancies between MLC leaf positions in the R&V database and those in the treatment plan. This usually occurs when the MLC leaf positions in the plan are generated automatically (MLC fit to a block), with some subsequent manual adjustments. After the plan has been saved and the parameters exported to the R&V database, subsequent interaction with the plan causes the MLC leaves to fit to the block, undoing the manual adjustments. The RTP‐Filter then compares the R&V database parameters that include the manual adjustments against the plan without the manual adjustments, and flags the errors. We have noticed an increase in the number of these subtle errors, mainly because our manual process of visual comparison of images of field shapes missed them, and visual comparisons were typically made against printouts which often represent the state of the planning data prior to the error.

We have also found errors in table positions for fields with the same isocenter. Since the entry of this information is not automated and the table positions have to be entered into each field one at a time, the potential for typographical errors increases. As a result, when the error occurs, there is usually one field whose table positions are different from those of all the other fields that share the isocenter. Earlier in our workflow development, therapists captured table positions at the LINAC instead of typing them in, but this unfortunately also captures table angles. When they forget to position the couch to the plan angle, they capture the incorrect angle. These errors were also caught by the filter. Prior to the implementation of the filter, these errors were caught by the therapists on treatment day, when they were unable to deliver the fields in an auto‐sequence. This inconvenience has been eliminated since we started using the filter.

Treatment fields that did not have assigned dose but that should have been part of the dose tracking have also been flagged, requiring the physicists to redistribute the assigned doses. TBI plans with wrong table positions have also been caught, as have other errors that are related to electron plans: applicator names are missing, bolus thicknesses are not indicated, MUs do not match, and collimator angles are incorrect. A large fraction of our errors are from electron plans, cone beam Y jaw values, and table positions. This is because these data involve direct human manipulation of the database values. Items that are imported into the database from LEX are less likely to have errors.

The benefits of using the filter are an improved rate of error detection and a more thorough check. The actual amount of time to do a chart check is about the same; however, automation has allowed us to check much more data. When we checked paper charts, for instance, we did not check all MLC leaf positions. We only checked the first and last open leaf pairs and compared the general shape in the R&V system with the Beam's eye view printouts from the plan. With our EQS, the plan quality review takes about 10 to 15 minutes, and the rest of the plan check takes about 10 and 25 minutes for 3DCRT and IMRT plans, respectively. However, if there are errors, the time required to complete the plan check is highly dependent on which processes in Fig. 5 need to be corrected. This additional time can range from 10 minutes to 2 hours. If the plan does not pass the IMRT virtual film QA, then an actual film measurement needs to take place, adding 2 hours to the process.

### B. Errors Missed by the RTP‐Filter

We have been using the RTP‐Filter in our clinic for the past two years. It has gone through some evolution as our workflows were modified and as errors were caught by processes other than the automated check of the RTP‐Filter. Because our plan check workflow requires several reviews of a plan (dosimetrist, physicist, therapist – see Fig. 5) before a patient is treated, conditions that were not anticipated in the implementation of the RTP‐Filter were still caught, and the logic for such conditions was then included in the application. Examples of such items are: 1) total number of treatment fractions in the prescription was changed in the R&V database but not in the plan; 2) the modality chosen from the R&V drop‐down list for the prescription did not match the energies of the treatment fields; 3) asymmetric cone beam field settings were reversed; 4) virtual wedge angles other than the commissioned one (60 degrees) were used; and 5) illegal monitor unit settings for virtual wedge were used. Since these changes were made, the filter has flagged these errors.

There are also some errors that occurred because new processes were implemented without modifying the plan check procedures. Fields planned to be “Gated” were not set up as gating fields in LANTIS, and the physicist and dosimetrist did not notice this. Non‐gated fields can not be delivered when the LINAC is set up for gating. This must be corrected prior to treatment, delaying the patient's treatment. The information about gated treatments does not get exported in the RTP‐Connect file, and the physicist must perform this “logical consistency” check by reading completely through the notes in the chart. There is no standard documentation requirement for this, and the physicians and dosimetrists tend to put them in a number of different locations. We have analyzed the exported data to find clues about fields that should be gated and concluded there was enough information to definitively check for gating for some patients but, for others, the information was incorrectly entered in the R&V database. We need a uniform documentation policy for gating so that the information is correctly entered and the appropriate data for logical consistency checking is exported.

Part of the difficulty with electronic plan checking is that there is usually no place in the planning system to indicate some types of information. We can not specify in the planning system that a beam is gated. The dosimetrist has to check a box in LEX to indicate this and, on a number of occasions, they have forgotten to do so. The same is true of dose rate. There are cases where, in fact, the maximum dose rate is not desired (e.g. pancreas and liver) and the physician specifies the dose rate in the patient's chart. Again, there is no field in the planning system to specify this, and the physicians do not always place this information in a part of the R&V database that gets exported in the RTP‐Connect file. The dosimetrist specifies the dose rate in LEX so that the dose rate is set correctly in the file that the R&V database imports. Physicists will recognize an error only if they are familiar with the typical dose rate settings for various types of treatments and they inspect the dose rate in each field.

There is also at least one item that is requested by the physician that is indicated in the patient's chart but that is not part of the treatment plan. This is the cone beam image‐processing filter, which is part of the cone beam protocol. The protocol filter setting is not stored in the R&V database; it is in the local database of the treatment machine. The cone beam field size and MU are part of the treatment plan, but they are not exported through LEX. Cone beam fields can be created only through the “Treatment Delivery” tab within Coherence Therapist (Siemens Medical Solutions, Concord, CA). This must be done prior to the initial plan check with the RTP‐Filter, to ensure that the field size and MU are set properly. This is especially important for those cases where the cone beam dose is included in the daily dose; it is not merely an imaging field but a treatment field as well. Hence, the treatment machine configuration for the cone beam can be checked against the treatment plan data using the RTP‐Filter, but the chosen protocol filter has to be verified by visual comparison between the selection on the local treatment machine and the patient's notes in the R&V database.

A less problematic error comes from the incorrect selection of the tolerance table for a particular plan. Fortunately, the tolerance table defaults to the strictest one and the error occurs when the dosimetrist forgets to choose a less strict tolerance table. Automatically determining tolerance tables for a given treatment plan is difficult because there is no standard exported data element that the RTP‐Filter can examine to make the unequivocal determination of the required tolerance table. Finally there is a class of errors that has minimal impact on treatment since they show up as documentation inconsistencies. For example, a dosimetrist may use a treatment plan template that populates field names with the standard gantry and couch angles. In the process of modifying the plan, the gantry or couch angles may be modified by 5 to 10 degrees, without changing field names. The RTP‐Filter does not attempt to parse the field name string into the angles for comparison with the actual angles in the field definition. There is no commonly accepted field naming convention adopted by the dosimetrists, so it would be harder to parse field name strings into meaningful data. We would be better off giving the field names generic names (e.g. beam 1) so that no logical inconsistencies occur.

The fact that the filter has not caught everything indicates the difficulty of anticipating errors from the very beginning. It is easy enough to write code that will compare parameters, but sometimes the data sources are deficient for the automated checking of some elements. The lack of standard procedures for documentation of important data makes it difficult to determine if other data elements are missing. It is also difficult to fully understand where related data elements occur and check that those relationships are logically consistent. Fortunately, the beauty of custom software is that we can improve it to capture these errors, and learn more about the details of our clinical workflow and the habits of people who interact with the data in these processes. As every clinic is different, it may be difficult to write automated plan checking software that is generic enough to be used by several institutions, unless clear standards about the most important processes are adopted by these institutions and vendors of commercial treatment planning systems and R&V databases.

It is important to note that several of the redundant check processes that do not use the RTP‐Filter were left in place (e.g. therapist review of plan data), and they played a significant role in preventing errors from escaping through the plan check workflow and incorrectly treating the patient. Automating a significant portion of a tedious process does not guarantee that it will catch all the errors. However, it allows the physicist to focus on the more difficult and subtle errors by automating straightforward but tedious data comparison tasks that are more prone to human error. The physicist still needs to be vigilant during the plan check, reading through the planning notes written by the dosimetrists and physicians in the R&V database and watching for possible logical inconsistencies. Monitoring the types of errors that have been missed but that do not have appropriate check routines implemented in the RTP‐Filter will teach the physicists which types of data relationships to examine.

### C. Errors Flagged by the System but Missed by People

In the early version of the user interface of the RTP‐Filter, error messages for many conditions were presented as dialog boxes. Unfortunately, a lot of other warnings that were not significant were also presented through dialog boxes. There was a tendency to ignore the messages and occasionally an important, valid error would be ignored. The interface was redesigned, replacing the message boxes with data report summaries. One is shown right after the file is read but prior to displaying the data. The second is shown after the user compares the RTP‐Connect file data with the data in Pinnacle. These steps reduce the number of dialog boxes presented to the user. Each of these reports presents a summary of the information that used to be displayed in the message boxes. Additionally, the background color of these reports turns red if there is an error (font color is white), green if everything is fine, and light yellow if there are warnings. Warnings are usually differences that are expected (e.g. a comparison of an IE file with an FE file should reveal table position differences and possibly setup note differences.)

Since the implementation of the new interface, there has only been one error that was flagged by the RTP‐Filter but ignored by a user. The RTP‐Filter log files confirmed that the error was recorded by the filter and the user who missed it was relatively inexperienced with the system. No automated system is foolproof, and while it may reduce the tedium and improve the checking of parameters, people must review the results and pay attention to the reports that the automated systems generate. Unless there is clear training provided as to the purpose and meaning of the various reports, errors can still occur.

### D. Lower Priority Errors

In the previous sections there are wish list items that are related to errors that have actually occurred that are being studied for ways to automate the checking. There are some errors that have not occurred that could be caught by an automated check, but they are of much lower priority due to the use of generic designations. For example, the LANTIS prescription “technique” drop down box has choices like “IMRT”, “4 field box”, “3 field”, “AP/PA”, and the generic “per plan”. The fact that the physician, dosimetrist, and physicist have reviewed and accepted the plan and that the parameters in the R&V database match those of the treatment plan minimizes the risk of a wrong treatment. The use of the technique descriptor in a prescription is somewhat antiquated, and is only useful in the context of a clinical setup (i.e. no treatment plan). Choosing the item “3 field” accidentally instead of “4 field box” for a case with a treatment plan will not cause the patient to be mistreated, since the treatment fields in the plan were approved, imported into the database, and checked. In this case, the error is one of documentation. In many cases, the error is avoided altogether by the use of the selection “per plan”.

### E. Hybrid Checks

When one does not have access to the data files or database that a planning system uses to generate parameters for treatment units, no direct data‐to‐data comparison can be performed electronically. However, the data from the R&V database can still be organized in a manner that facilitates the comparison. Our initial comparison of the printed FASTPLAN data with the LANTIS database was difficult, since repetitive data was not conveniently grouped. Checks were done on a field‐by‐field basis. The RTP‐Filter was modified to analyze the LANTIS data and present a single report that grouped fields according to isocenter. Additionally, the format of the report is very similar to that of the FASTPLAN printout. Finally, the filter can still perform the logical consistency checks on the LANTIS database. This has increased the efficiency with which we check SRS plans, even though we still perform a visual comparison of plan data to R&V data. An estimated reduction in time for plan checking is about 30%.

### F. Recommendations

Automated plan checking would benefit from consistent documentation practices so that the data is found in well‐defined locations. Vendors of treatment planning systems and R&V databases should adopt standards that define the minimum required data set^(6)^ ^,^ ^(9)^ and the locations where these should be found for the purpose of electronic data interchange and data integrity checks. Table I indicates the parameters that we check at our clinic, and in order to fully document items in the exported files that are missing from the planning system, we use an in‐house application, LEX. There are items that we still check visually because the data is not available in the exported files. We are currently testing a modification to the LANTIS Export Manager utility (an in‐house application written by Author 1 in visual basic.net) that will read the LANTIS database with SQL queries via Open Database Connectivity (ODBC) in order to write a text file that contains all the patient's notes. This occurs at the time that the export manager reads the RTP‐Connect file that was exported out of LANTIS. This will enable us to modify the RTP‐Filter to search for key words in the notes. The reliability of such automated checks will still depend on adopting standards for entering notes, and we are developing a note template for treatment planning information.

The human interaction with the complete system that comprises the planning system, the R&V database, and the planning check workflow, should be well focused, limiting the entry point of human‐determined information to one location. When a physician requests a treatment to be gated, that request should be entered in the planning system instead of in the R&V database. The written prescription should be written in the planning system as well, not just for the purpose of MU calculations but also to reduce the repetitive entry of information in multiple locations. Note that for IMRT, the final prescription in Pinnacle is MU based, while the physician's prescription is dose based (DVHs). Such DVH prescriptions could be easily captured in the planning system.

The logical consistency checks that were programmed in the RTP‐Filter generally operate on a set of data that is contained in one location, normally the R&V database. Logical consistency checks can be a function of the database application, where the database cross‐checks the relationships among its data. Such logical checks should also be done within the treatment planning system and, in fact, LEX performs some rudimentary checks of the planning data before it exports the RTP‐Connect file. The RTP‐Filter performs a few logical checks that require reading the data from both the R&V database and the planning system. Migrating all the data entry functions to the planning system (for items in Table I) would allow a complete logical consistency check within one location.

As new treatment techniques are developed, we must also define procedures for documenting technique‐specific information. If planning systems provided user‐definable comment fields for documenting technique parameters, then there would be a well defined location that applications could read in order to automate parameter checking. This could be an interim solution while standards are modified to accommodate advances in the field.

For commercial systems that claim the full integration of the planning database as part of the R&V database, data integrity checks would be reduced to checking the state of the database with snapshots of a known good state. However, prior to defining these snapshots, logical consistency checks must still be performed. Single database or integrated database systems are still subject to the entry of information with multiple relationships, and these relationships must be verified. Additionally, the claims of full integration must still be verified during the acceptance test procedure.

Finally, our ability to prevent treatment errors also depends on redundant check processes, so that multiple fault failures are required before a treatment error occurs. Automating the parameter checking does not mean one can remove the parameter checks performed in other portions of the workflow, especially when one is in the test phase of the automation. The definition of, and adherence to, the plan check process is just as important as the automated data integrity and logical consistency checks. When multiple applications access the same database, the change of data through one application can change secondary information presented by other applications, and the plan check process has to define what applications should be reviewed after database changes are made. This is also the reason why our workflow requires that the R&V data are compared to the plan, rather than the file exported from the plan. The file exported from the plan is a snapshot of the state of the plan prior to the export, but it is possible that deviations from the workflow may result in interactions with the plan that may change its data.

## V. CONCLUSIONS

Automated checking of plan parameters reduces error rates by allowing a more thorough check of the R&V database and the treatment plan. It greatly reduces the tedious one‐to‐one data correspondence checks and formalizes the checks for logical consistency. As long as physicists receive the proper training and follow the plan check process, running the comparisons and monitor unit calculations with the filter ensures that all physicists perform the same checks. The number of manual checks has been greatly reduced, minimizing the risk that a physicist will forget to check an item. A number of recommendations to treatment planning system and R&V vendors would help improve the ability to perform the automated checking and reduce the errors in the system. The advantage of being paperless is that data can be interchanged and checked electronically, but vendors should adopt standards to improve the quality of the plan check process. Even with standards in place, there must be mechanisms to account for variations in clinical practice. We are fortunate that we have the resources and the knowledge to develop independent, custom software that is specifically designed for our clinic.

However, as automated checking software is being implemented, it should be part of a redundant planning check process so that errors that escape detection with the automated check can still be caught by another check process further downstream in the workflow. Hence, the concept of an Electronic QA System includes not only the applications that automate the checks but also the workflow that ensures that the planning check process is done correctly. We should not disengage ourselves from the plan check process and implicitly rely on automated systems – they should be considered as assistants who reduce the drudgery of a process to produce results that are still subject to our final review. This is true even for systems that claim to use an integrated database for planning and R&V, since it is still possible to introduce logical inconsistencies among the data.
